# Prognostic value of admission serum magnesium and systemic inflammation indices in septic ICU patients aged 85 years and older: A retrospective observational study

**DOI:** 10.1097/MD.0000000000044979

**Published:** 2025-10-17

**Authors:** İrem Akin Şen

**Affiliations:** a Department of Internal Medicine, Division of Medical Intensive Care, Faculty of Medicine, Mersin University, Mersin, Turkey.

**Keywords:** elderly patients, hypermagnesemia, ICU mortality, inflammatory biomarkers, sepsis

## Abstract

This study aimed to investigate the prognostic value of admission serum magnesium levels and systemic inflammatory indices, including C-reactive protein (CRP), neutrophil-to-lymphocyte ratio, systemic immune-inflammation index (SII), and multiple inflammation index, for predicting key clinical outcomes in critically ill patients with sepsis aged ≥ 85 years. This retrospective observational study was conducted to evaluate the prognostic value of admission serum magnesium and systemic inflammatory indices in critically ill patients with sepsis aged ≥ 85 years. Patients were stratified by serum magnesium levels into hypomagnesemia (<1.8 mg/dL), normomagnesemia (1.8–2.4 mg/dL), and hypermagnesemia (>2.4 mg/d). The prognostic performance for intensive care unit (ICU) mortality, 28-day mortality, mechanical ventilation requirement, and prolonged ICU stay was assessed using receiver operating characteristic curve analysis and multivariable Cox proportional hazards models. Among 381 patients aged ≥ 85 years (mean age 89.2 ± 3.2; 59.3% male) admitted to the ICU with sepsis, the ICU and 28-day mortality rates were 33.1% and 37.8%, respectively. Hypermagnesemia (>2.4 mg/dL) was observed in 18.9% of patients and was associated with higher sequential organ failure assessment (sequential organ failure assessment) scores (9.2 ± 3.1), CRP (129.7 ± 84.6 mg/L), neutrophil-to-lymphocyte ratio (15.2 ± 10.8), and multiple inflammation index (564.3 × 10³), with an ICU mortality of 52.8% (*P* = .001) and 28-day mortality of 61.1% (*P* < .001). In ICU patients with sepsis aged ≥ 85 years, hypermagnesemia at admission was a strong and independent predictor of mortality.

## 1. Introduction

The incidence of sepsis in extremely elderly patients (≥85 years) is rising due to demographic aging.^[1]^ These patients are highly vulnerable due to frailty, comorbidities, and limited physiological reserve.^[2]^ Magnesium plays critical roles in immune regulation and inflammation. Both hypo- and hypermagnesemia are linked to poor outcomes in critical illness, yet data in the extremely old are scarce. Inflammatory indices such as NLR, PLR, SII, and MII are established prognostic markers, but their interaction with magnesium in this age group remains unclear. This study investigates the independent and combined prognostic value of serum magnesium and inflammatory indices in septic ICU patients aged ≥ 85 years.

Magnesium, an abundant intracellular divalent cation, is involved in more than 300 enzymatic reactions and plays pivotal roles in immune regulation, vascular tone, energy metabolism, and inflammation modulation. Despite its biological relevance, Mg is frequently overlooked in the prognostic assessment of patients with sepsis. Both hypomagnesemia and hypermagnesemia are associated with deleterious outcomes in critically ill patients, including increased risk of mechanical ventilation, prolonged ICU stay, and mortality. While magnesium derangements are common in elderly individuals due to altered renal handling, polypharmacy, and malnutrition, their prognostic significance in sepsis, particularly among the extremely old, remains insufficiently characterized.^[3,4]^ Recent high-powered observational studies have suggested that elevated serum magnesium levels at ICU admission are independently associated with increased short-term mortality in patients with sepsis.^[4,5]^ This association appears to reflect not only the direct physiological effects of magnesium on cardiovascular and neuromuscular functions but also its intricate interplay with the systemic inflammatory response. Concurrently, a growing body of literature emphasizes the utility of composite inflammatory indices, such as the neutrophil-to-lymphocyte ratio (NLR), platelet-to-lymphocyte ratio (PLR), systemic immune-inflammation index (SII), and multiple inflammation index (MII), as robust markers of sepsis-related prognosis in geriatric cohorts.^[6–11]^ Nevertheless, current evidence addressing the interaction between serum magnesium levels and systemic inflammation, as reflected by these indices, is limited and fragmented. Furthermore, the prognostic implications of magnesium levels in relation to concrete clinical outcomes, such as length of ICU and hospital stay, need for mechanical ventilation, and in-hospital mortality, have not been systematically evaluated in elderly patients with sepsis. This knowledge gap is particularly critical given the unique immunosenescence profile and increased baseline vulnerability of this age group.

This study aimed to investigate the prognostic relevance of serum magnesium concentrations at admission in critically ill patients aged ≥ 85 years with sepsis. Specifically, we evaluated the independent and combined predictive values of magnesium levels and systemic inflammatory indices for key clinical outcomes. By focusing on an extremely elderly population, this study sought to refine early risk-stratification strategies and to highlight the utility of routinely available biomarkers in geriatric care.

## 2. Material and methods

### 2.1. Study design and setting

This retrospective observational study was conducted in the medical intensive care unit (ICU) of a tertiary academic center in Turkiye. The study protocol was reviewed and approved by the institutional ethics committee, and all procedures were performed in accordance with the principles of the Declaration of Helsinki and relevant national regulations (June 17, 2025, app no: 7807789/050.01.04/3134538). Data were collected over a 5-year period between January 2019 and December 2024.

### 2.2. Study population

The study included patients aged ≥ 85 years who were admitted to the ICU with a diagnosis of sepsis, defined in accordance with the Sepsis-3 criteria, as a documented or suspected infection accompanied by an acute increase of ≥ 2 points in the Sequential Organ Failure Assessment (SOFA) score. Eligibility was limited to patients whose admission serum magnesium concentrations and baseline inflammatory markers were available in the database. Exclusion criteria were active malignancy, recent trauma, postoperative admission status, prior magnesium supplementation before ICU admission, ICU readmission during the same hospitalization, and incomplete clinical or laboratory data. After applying these criteria, 381 patients were finally included in the analysis.

### 2.3. Data collection

Patient data were extracted from the institutional electronic medical records. The following variables were recorded at the time of ICU admission: age, sex, Clinical Frailty Scale (CFS) score, Glasgow Coma Scale (GCS) score, SOFA score, comorbid conditions (hypertension, diabetes mellitus, coronary artery disease, congestive heart failure, cerebrovascular disease, chronic kidney disease, and chronic obstructive pulmonary disease), and infection focus (pneumonia, urinary tract infection, intra-abdominal infection, or other/unknown). Laboratory parameters included serum magnesium (mg/dL), C-reactive protein (CRP, mg/L), white blood cell count (×10³/μL), lactate (mg/dL), serum creatinine (mg/dL), sodium (mmol/L), albumin (g/dL), hemoglobin (g/dL), and platelet count (×10³/μL). Inflammatory indices were calculated as follows: NLR, PLR, systemic immune-inflammation index (SII; platelet count × neutrophil count/lymphocyte count), and MII (defined as SII × C-reactive protein [CRP]). Frailty was defined as a CFS score of ≥ 5. The use of vasopressors during the ICU stay was also recorded.

### 2.4. Outcomes

The primary outcome measures were the ICU and in-hospital mortality rates. Secondary outcomes included length of ICU stay, total length of hospital stay, and duration of invasive mechanical ventilation among ventilated patients.

### 2.5. Group definitions and statistical analysis

Patients were stratified into 3 groups based on admission serum magnesium levels: hypomagnesemia (<1.8 mg/dL), normomagnesemia (1.8–2.4 mg/dL), and hypermagnesemia (>2.4 mg/dL), according to widely accepted clinical thresholds. Continuous variables were summarized as mean ± standard deviation and median with interquartile range (IQR), and categorical variables were reported as absolute numbers and percentages. Distribution normality was assessed using the Shapiro–Wilk test. Intergroup comparisons were conducted using the Kruskal–Wallis test or one-way analysis of variance (ANOVA) for continuous variables, depending on the distribution characteristics, and Pearson chi-square or Fisher exact tests for categorical variables. Statistical significance was set at *P* < .05. All analyses were performed using IBM SPSS Statistics for Windows, version 26.0 (IBM Corp., Armonk).

## 3. Results

A total of 381 patients aged ≥ 85 years with sepsis admitted to the ICU were included in the final analysis. The mean age was 89.2 ± 3.2 years, and 59.3% of the patients were male. A CFS score of ≥ 5 was observed in 70.9% of the patients. The most frequent comorbidities were hypertension (70.9%), congestive heart failure (33.9%) and diabetes mellitus (26.8%). Pneumonia (47.2%) and urinary tract infections (30.2%) were the most common foci of infection in this study. The mean SOFA score was 8.0 ± 3.0, and the median GCS score was 12 (IQR: 3–14). The mean serum magnesium level at ICU admission was 2.0 ± 0.5. The median values for the inflammatory indices were as follows: NLR = 8.0, PLR = 172, SII = 1557, and MII, 101,792. Vasopressor use was recorded in 91.1% of patients. The median ICU and hospital lengths of stay were 6 and 7 days, respectively. ICU and 28-day mortality rates were 33.1% and 37.8%, respectively (Table S1, Supplemental Digital Content, https://links.lww.com/MD/Q284).

Patients were stratified into 3 groups based on their serum magnesium concentrations at admission: hypomagnesemia (<1.8 mg/dL), normomagnesemia (1.8–2.4 mg/dL), and hypermagnesemia (>2.4 mg/dL). Group comparisons showed that serum magnesium, CRP, NLR, PLR, SII, and MII levels differed significantly across magnesium strata (*P* < .05). The hypermagnesemia group had the highest mean values for serum magnesium (3.0 ± 1.7 mg/dL), CRP (129.7 ± 84.6 mg/L), NLR (15.2 ± 10.8), PLR (328.7 ± 205.9), SII (4324 ± 3211), and MII (564.3 ± 337.4 × 10³). The SOFA score also differed significantly between the groups (*P* = .01), being highest in the hypermagnesemia group. Creatinine and sodium levels were significantly elevated in patients with hypermagnesemia (*P* < .001 and *P* = .03, respectively). ICU and 28-day mortality rates were the highest in the hypermagnesemia group (52.8% and 61.1%, respectively; *P* = .001 and *P* < .001, respectively) (Table S2, Supplemental Digital Content, https://links.lww.com/MD/Q284).

Receiver operating characteristic (ROC) analysis revealed that serum magnesium predicted ICU mortality with an AUC (area under the curve) of 0.72, using an optimal cutoff value of 2.04 mg/dL. Among inflammatory markers, CRP (AUC = 0.66), NLR (AUC = 0.70), SII (AUC = 0.64), and MII (AUC = 0.68) showed moderate discrimination. The combined models, including magnesium with CRP, NLR, SII, and MII, enhanced the predictive performance, with magnesium + CRP yielding the highest AUC (0.76 for ICU mortality. Similarly, for the 28-day mortality, magnesium + CRP and magnesium + MII showed AUC values of 0.74. For mechanical ventilation, magnesium alone had an AUC of 0.74, and magnesium + CRP had the highest performance (AUC = 0.76). All individual and combined markers showed poor discrimination in predicting prolonged ICU stay, with maximum AUC values below 0.61 (Table S3, Supplemental Digital Content, https://links.lww.com/MD/Q284).

Multivariable Cox proportional hazards models were constructed for the 4 binary outcomes. For mechanical ventilation, magnesium > 2.4 mg/dL (HR = 1.76, *P* = .004), CRP ≥ 107 mg/L (HR = 1.41, *P* = .039), NLR ≥ 7 (HR = 1.37, *P* = .044), SII ≥ 1908 (HR = 1.35, *P* = .048), and MII ≥ 92 (HR = 1.39, *P* = .032) were independently associated with an increased risk. For prolonged ICU stay, only the SOFA score was statistically significant (hazard ratio [HR] = 1.09, *P* = .014). For ICU mortality, magnesium > 2.4 mg/dL (HR = 1.90, *P* = .001), CRP ≥ 107 mg/L (HR = 1.60, *P* = .015), NLR ≥ 7 (HR = 1.45, *P* = .028), SII ≥ 1908 (HR = 1.48, *P* = .014), and MII ≥ 92 (HR = 1.44, *P* = .022) were significant predictors. The same variables were also independently associated with 28-day mortality, with identical HRs and p values (Table S4, Supplemental Digital Content, https://links.lww.com/MD/Q284).

## 4. Discussion

In this retrospective cohort study of ICU patients with sepsis aged ≥ 85 years, serum magnesium level at admission emerged as a significant prognostic biomarker. Patients with mild hypermagnesemia on admission had substantially higher ICU and 28-day mortality rates than those with normal magnesium levels. This finding aligns with recent large-scale studies linking elevated serum magnesium levels to worse outcomes in sepsis.^[4,12,13]^ For instance, an international multicenter study by Li et al demonstrated a progressively increased 28-day mortality risk with increasing magnesium levels, validating serum magnesium as an independent risk factor for sepsis-related mortality across diverse populations.^[4]^ Similarly, critical care data have shown that hypermagnesemia is associated with dramatically higher mortality, highlighting the adverse prognostic impact of high magnesium levels across age groups.^[14]^ Our study extends these observations to extremely elderly patients, a demographic in which sepsis poses a unique challenge. The fact that admission magnesium > 2.4 mg/dL conferred nearly twice the hazard of death, even after adjusting for other markers, underscores that serum magnesium level provides independent prognostic information beyond conventional indices. In practical terms, this suggests that an often-overlooked electrolyte measurement could serve as an early warning marker for geriatric sepsis, identifying high-risk patients upon ICU admission. Given that magnesium testing is rapid and routine, its incorporation into initial risk assessment could enhance clinical vigilance in this vulnerable cohort.

The association between elevated magnesium levels and poor outcomes likely reflects the underlying pathophysiology of severe sepsis. Notably, patients in the hypermagnesemia group presented with significantly higher CRP levels and immune cell–derived indices (NLR, SII, and MII) than normomagnesemic patients. They also had more organ dysfunction, as evidenced by the higher SOFA scores and creatinine levels in the hypermagnesemia group. This finding suggests that magnesium elevation coincides with an intense inflammatory response and acute organ failure in patients with sepsis. Magnesium homeostasis is intimately associated with renal function, and acute kidney injury and impaired clearance in critical illness can lead to magnesium accumulation.^[14]^ Indeed, previous analyses have noted that hypermagnesemic patients are more likely to have coexisting acute kidney injury and hepatic dysfunction, supporting the notion that serum magnesium serves as a surrogate marker for organ failure severity in sepsis.^[13,15,16]^ In addition to being a bystander, magnesium may actively interact with the inflammatory pathways. Experimental and clinical studies have predominantly highlighted the consequences of low magnesium levels. For example, magnesium deficiency triggers leukocyte and macrophage activation, cytokine release, and elevated acute-phase reactants such as CRP.^[14]^ In chronic contexts, lower magnesium status correlates with heightened systemic inflammation and oxidative stress.^[14]^ Paradoxically, among our acutely ill population, those with the highest magnesium levels also had the highest levels of inflammatory markers. This likely reflects the fact that hypermagnesemia in sepsis is not a benign, anti-inflammatory state but rather a marker of critical derangements that simultaneously drive inflammation. It is plausible that once magnesium levels rise above the normal range because of these derangements, any direct anti-inflammatory effects of magnesium are overwhelmed by the severity of the illness. Moreover, extremely old patients often have a baseline proinflammatory milieu owing to immunosenescence and comorbidities.^[1]^ For instance, it is frequently elevated in the elderly owing to chronic conditions. In such patients, an acute spike in magnesium levels may serve as an additional red flag for decompensation that pure inflammatory markers cannot capture.^[1]^ Therefore, our findings underscore a complex interplay: serum magnesium in sepsis reflects both the inflammatory burden and degree of organ dysfunction, particularly in the elderly, who have diminished physiological reserves.

A key question is how serum magnesium levels compare with established inflammatory biomarkers in predicting outcomes. In our cohort, magnesium at admission alone showed fair discriminative ability for mortality, comparable to or better than single inflammatory indices. This level of discrimination is notable; for context, elevated composite indices such as the SII have been associated with a higher sepsis mortality risk, but their predictive power in older patients can be modest.^[17]^ The neutrophil–lymphocyte ratio and related indices are well-established prognostic markers in sepsis and critical illness; however, no single marker is sufficient for isolation.^[17]^ Our findings suggest that serum magnesium level is on par with these conventional biomarkers in terms of prognostic relevance, capturing risks that might otherwise require more complex scoring. This resonates with prior clinical observations outside the sepsis realm. For example, Tan et al reported that even high-normal magnesium levels were associated with increased 30-day mortality in critically ill cardiac patients.^[5]^ Their study in an acute myocardial infarction ICU cohort found an adjusted hazard of death 1.3–to 1.6-fold higher in patients with magnesium in the upper-normal or mildly elevated range.^[5]^ Taken together with our results, it is evident that subtle elevations in magnesium levels can have prognostic significance across different critically ill populations.

Importantly, we demonstrated that combining magnesium with inflammatory markers improved predictive performance. Integrated models achieved higher AUC values than either marker alone; for example, magnesium + CRP yielded an AUC of ~0.76 for ICU mortality, the highest of all the combinations tested. This incremental gain in discrimination, although moderate, was clinically meaningful. This suggests that magnesium levels and inflammatory indices provide complementary prognostic information. Biologically, this makes sense: CRP and cell-count ratios reflect the magnitude of the immune response, whereas serum magnesium may indicate the degree of metabolic and organ dysfunction, 2 distinct but intersecting facets of critical illness severity. Combined assessment captures a broader snapshot of a patient’s condition. The fact that our multivariable Cox models found magnesium, CRP, NLR, SII, and MII to be independent mortality predictors further reinforces the fact that no single parameter subsumes the effects of others. Each marker likely flags a different dimension of risk and its convergence identifies patients at the highest risk of developing HF. From a practical standpoint, this additive prognostic value is promising. All of these markers are inexpensive and readily available upon admission. Incorporating magnesium into risk stratification along with inflammatory markers could enhance the early identification of high-risk elderly patients with sepsis who might benefit from aggressive monitoring or interventions. This is analogous to the multi-biomarker approaches used in sepsis and other ICU conditions, where combinations of variables outperform individual metrics in terms of prognostic accuracy.^[7]^ Our data support moving beyond a siloed evaluation of either electrolytes or inflammation, and toward an integrated model in the ICU admission workup for patients with stroke.

Our findings have several clinical implications for managing patients with sepsis of advanced age. First, serum magnesium levels, which are often not central to sepsis scoring systems, should be recognized as significant indicators of illness severity in older adults. Hypermagnesemia on arrival may alert clinicians to occult organ failure and heightened risk of deterioration, even if traditional scores or vital signs are equivocal in this age group. Second, the strong association of magnesium with mortality and mechanical ventilation suggests that magnesium levels could inform prognostic discussions and decisions about the intensity of care (e.g., flagging patients who might need early respiratory support or renal replacement therapy). Third, the enhanced predictive value observed when magnesium was combined with inflammatory markers highlights the potential of an additive risk assessment strategy. Rather than relying on a single laboratory value, geriatric sepsis care may benefit from simple composite indicators; for instance, a combined magnesium-and-CRP threshold could be used to triage octogenarian patients at the highest risk of death or prolonged ICU needs. This approach is supported by the literature showing improved outcomes when multifactorial assessments are applied to older ICU patients. Finally, while our study focused on prognostication, it also prompts consideration of whether targeted management of magnesium abnormalities could influence the outcomes. Emerging evidence suggests that magnesium supplementation in critically ill patients is associated with reduced mortality, and ongoing research is examining its immunomodulatory therapeutic potential.^[18–20]^ Although treating hypermagnesemia is more challenging, recognizing it as a risk marker is the first step in its management. In summary, serum magnesium should be used alongside lactic acid and traditional inflammatory biomarkers in the toolbox for sepsis risk assessment in elderly patients. By acknowledging the prognostic relevance of magnesium and leveraging it in combination with other indices, critical care teams can refine their early risk stratification and improve the management of this growing population of vulnerable patients with sepsis. In practical terms:

- Hypomagnesemia is often corrected in critical care, but optimal treatment thresholds for extremely elderly septic patients remain undefined.
- Hypermagnesemia management focuses on treating the underlying cause, optimizing renal clearance, and initiating renal replacement therapy when indicated.
- Until interventional studies provide more evidence, hypermagnesemia should primarily be considered a prognostic marker rather than a direct therapeutic target.

## 5. Study strengths and limitations

This study represents one of the largest evaluations of admission serum magnesium levels and systemic inflammation indices in ICU patients with sepsis aged ≥ 85 years. Including this vulnerable geriatric cohort strengthens its clinical relevance and generalizability to aging populations worldwide. The analysis of magnesium and 4 inflammatory biomarkers (CRP, NLR, SII, and MII) allowed for comprehensive risk stratification. ROC curve analysis and multivariate Cox regression models ensured robust statistical validation of the findings.

However, the retrospective, single-center design limits causal inference and may introduce a selection bias. Unmeasured confounders, such as pre-admission magnesium supplementation or baseline nutritional status, were not accounted for in this study. Additionally, changes in magnesium and inflammatory indices during ICU stay were not evaluated, which may have provided insights into temporal risk evolution. Although the thresholds were derived using ROC analysis, prospective external validation is required before their clinical implementation. Additionally, the study did not include a younger patient comparison group, which limits the generalizability of findings beyond the ≥ 85-year-old population. Future studies should assess whether these associations hold in younger cohorts.

## 6. Conclusion

In critically ill patients aged ≥ 85 years with sepsis, admission hypermagnesemia (>2.4 mg/dL) was independently associated with increased ICU admission and 28-day mortality rates. Elevated magnesium levels are closely correlated with systemic inflammation and organ dysfunction, reinforcing their potential as surrogate biomarkers of disease severity.

Serum magnesium levels demonstrated prognostic performance comparable to well-established inflammatory indices and further improved predictive accuracy when combined with CRP, NLR, SII, and MII. These findings support the integration of magnesium into early risk-stratification models for geriatric patients with sepsis.

Routine assessment of serum magnesium levels on ICU admission may aid in the early identification of high-risk elderly patients, especially when interpreted in conjunction with systemic inflammatory markers. Simple, cost-effective, and readily accessible magnesium is a valuable, yet often overlooked, biomarker with practical utility in geriatric critical care.

## Author contributions

**Conceptualization:** İrem Akin Şen.

**Data curation:** İrem Akin Şen.

**Formal analysis:** İrem Akin Şen.

**Funding acquisition:** İrem Akin Şen.

**Investigation:** İrem Akin Şen.

**Methodology:** İrem Akin Şen.

**Project administration:** İrem Akin Şen.

**Resources:** İrem Akin Şen.

**Software:** İrem Akin Şen.

**Supervision:** İrem Akin Şen.

**Validation:** İrem Akin Şen.

**Visualization:** İrem Akin Şen.

**Writing – original draft:** İrem Akin Şen.

**Writing – review & editing:** İrem Akin Şen.

## Supplementary Material


