# Supplementary material for: Prognostic value of admission serum magnesium and systemic inflammation indices in septic ICU patients aged 85 years and older: A retrospective observational study
Source: Medicine (Baltimore). 2025 Oct 17;104(42):e44979. doi: 10.1097/MD.0000000000044979 (PMC12537170; doi:10.1097/MD.0000000000044979)
Supplement: Supplementary file 1 [file medi-104-e44979-s001.pdf]

Supplementary Table 1: Baseline characteristics of septic ICU patients aged ≥85 years

Characteristic	All patients (N = 381)
**Demographic**	
Age, years	89.2 ± 3.2; 89 (87–91)
Male sex	226 (59.3%)
Frailty (CFS ≥5), n (%)	270 (70.9%)
**Comorbidities**	
Hypertension, n (%)	270 (70.9%)
Diabetes mellitus, n (%)	102 (26.8%)
Coronary artery disease, n (%)	97 (25.5%)
Congestive heart failure, n (%)	129 (33.9%)
Cerebrovascular disease (stroke), n (%)	29 (7.6%)
Chronic kidney disease, n (%)	37 (9.7%)
Chronic obstructive pulmonary disease	74 (19.4%)
**Clinical**	
GCS, points	9.5 ± 4.9; 12 (3–14)
SOFA score, points	8.0 ± 3.0; 8 (6–10)
**Infection focus, n (%)**	
– Pneumonia	180 (47.2%)
– Urinary tract infection	115 (30.2%)
– Intra-abdominal infection	50 (13.1%)
– Other/unknown^	36 (9.4%)
**Laboratory**	
Magnesium (Mg, mg/dL)	2.0 ± 0.5; 1.9 (1.8–2.3)
C-reactive protein (CRP, mg/L)	93.7 ± 85.1; 70 (25–141)
Neutrophil–lymphocyte ratio (NLR)	12.5 ± 13.6; 8.0 (5.0–14.9)
Platelet–lymphocyte ratio (PLR)	263 ± 355; 172 (111–301)
Systemic immune-inflammation index (SII)	2859 ± 1533; 1557 (819–3194)
Multiple inflammation index (MII) *	353,940 ± 169,994; 101,792 (30,423–286,249)
White blood cell count (WBC, ×10^3/µL)	12.6 ± 7.3; 11.0 (7.7–15.7)
Lactate (mg/dL)	25.3 ± 20.9; 15.8 (10.4–33.8)
Sodium (mmol/L)	139.8 ± 9.5; 139 (134–143)
Creatinine (mg/dL)	1.7 ± 1.2; 1.3 (0.8–2.2)
Albumin (g/dL)	2.7 ± 0.6; 2.8 (2.3–3.1)
Hemoglobin (g/dL)	10.5 ± 2.2; 10.2 (9.0–11.9)
Platelet count (×10^3/µL)	216 ± 124; 198 (138–269)

**Outcomes**	
Vasopressor use, n (%)	347 (91.1%)
ICU length of stay (days)	9.0 ± 7.2; 6 (4–12)
Hospital length of stay (days)	9.9 ± 8.3; 7 (4–14)
Duration of mechanical ventilation (days) ‡	9.3 ± 8.0; 7 (3–12)
ICU mortality, n (%)	126 (33.1%)
28-day mortality, n (%)	144 (37.8%)

All continuous variables are presented as mean ± SD and median (IQR), and categorical variables are presented as numbers (percentages).

^: less common infection foci, including catheter-related bloodstream infection, skin and soft tissue infection, biliary tract infection, infective endocarditis, and cases with an undetermined source.

‡ Patients who required invasive mechanical ventilation (n = 20). CFS, Clinical Frailty Scale; SOFA, Sequential Organ Failure Assessment; GCS, Glasgow Coma Scale; CRP, C-reactive protein; NLR, neutrophil-to-lymphocyte ratio; PLR, platelet-to-lymphocyte ratio; SII, systemic immune-inflammation index (platelet count × neutrophil count/lymphocyte count); MII, multiple inflammation index (SII × CRP); WBC, white blood cell; ICU, intensive care unit; LOS, length of stay; MV, mechanical ventilation.

Supplementary Table 2. Comparison of patient characteristics and outcomes by magnesium status in septic ICU patients aged ≥85 years

**Characteristic**	**Hypomagnesemia (n = 105)**	**Normomagnesemia (n = 203)**	**Hypermagnesemia (n = 72)**	***p*-value**
**Demographic**				
Age, years	89.2 ± 3.1; 89 (87–91)	89.2 ± 3.1; 89 (87–91)	89.0 ± 3.3; 88 (86–91)	0.88
Male sex, n (%)	62 (59.0%)	126 (62.1%)	37 (51.4%)	0.28
**Comorbidities**				
Hypertension, n (%)	81 (77.1%)	139 (68.5%)	46 (63.9%)	0.53
Diabetes mellitus, n (%)	27 (25.7%)	58 (28.6%)	16 (22.2%)	0.56
Coronary artery disease, n (%)	18 (17.1%)	60 (29.6%)	19 (26.4%)	0.06
Congestive heart failure, n (%)	34 (32.4%)	72 (35.5%)	23 (31.9%)	0.80
Cerebrovascular disease (stroke), n (%)	12 (11.4%)	11 (5.4%)	6 (8.3%)	0.16
Chronic kidney disease, n (%)	16 (15.2%)	11 (5.4%)	10 (13.9%)	0.01
Chronic obstructive pulmonary disease, n (%)	28 (26.7%)	31 (15.3%)	14 (19.4%)	0.06
**Clinical**				
GCS, points	8.0 ± 5.0; 3 (3–13)	10.2 ± 4.8; 12 (3–14)	12.6 ± 3.4; 14 (11–14)	0.02
SOFA score, points	7.3 ± 2.7; 7 (5–9)	8.0 ± 2.9; 8 (6–10)	9.2 ± 3.1; 9 (7–12)	0.01
Infection focus, n (%) – Pneumonia	50 (47.6%)	96 (47.3%)	34 (47.2%)	0.99
– Urinary tract infection	32 (30.5%)	61 (30.0%)	22 (30.6%)	0.99
– Intra-abdominal infection	13 (12.4%)	27 (13.3%)	9 (12.5%)	0.99
– Other/unknown	10 (9.5%)	19 (9.4%)	7 (9.7%)	0.99
**Laboratory**				
Magnesium (mg/dL)	1.6 ± 0.2; 1.6 (1.1–1.7)	2.0 ± 0.2; 2.0 (1.9–2.1)	3.0 ± 1.7; 2.7 (2.5–3.7)	< 0.001
CRP (mg/L)	76.4 ± 58.2; 62 (24–108)	89.1 ± 72.5; 70 (28–128)	129.7 ± 84.6; 104 (61–178)	**0.009**
NLR	8.3 ± 6.1; 6.2 (3.9–10.7)	11.5 ± 9.3; 8.5 (4.8–14.9)	15.2 ± 10.8; 11.6 (7.1–19.3)	**0.011**
PLR	211.8 ± 149.2; 175 (108–276)	263.2 ± 187.5; 200 (132–325)	328.7 ± 205.9; 278 (184–393)	**0.023**

SII	2,102 ± 1,573; 1,405 (870–2,590)	3,128 ± 2,014; 1,782 (1,030–3,984)	4,324 ± 3,211; 2,490 (1,614–6,020)	**0.015**
MII (×10³)	181.3 ± 122.8; 99.1 (35.4–235.0)	296.6 ± 164.2; 180.9 (72.8–418.2)	564.3 ± 337.4; 398.6 (174.2–763.9)	**<0.001**
White blood cell count (×10^3/µL)	12.2 ± 6.5; 11.2 (7.7–15.2)	13.3 ± 13.0; 10.4 (7.5–15.5)	14.7 ± 9.9; 12.1 (8.4–17.3)	0.31
Lactate (mmol/L)	24.0 ± 20.0; 15.0 (10.0–33.0)	26.0 ± 21.0; 16.0 (10.0–34.0)	24.5 ± 22.0; 15.0 (10.0–33.0)	0.93
Sodium (mmol/L)	137 ± 15; 137 (134–141)	139 ± 8; 139 (134–142)	144 ± 14; 140 (135–148)	0.03
Creatinine (mg/dL)	1.5 ± 1.2; 1.2 (0.8–1.6)	1.7 ± 2.0; 1.2 (0.8–2.2)	2.3 ± 1.4; 1.9 (1.2–3.2)	< 0.001
Albumin (g/dL)	2.7 ± 0.6; 2.8 (2.3–3.1)	2.7 ± 0.6; 2.8 (2.3–3.1)	2.6 ± 0.6; 2.6 (2.2–3.0)	0.31
Hemoglobin (g/dL)	10.3 ± 2.2; 10.0 (9.0–11.9)	10.9 ± 2.2; 10.2 (9.0–11.9)	11.0 ± 2.3; 10.9 (9.0–12.8)	0.22
Platelet count (×10^3/µL)	239 ± 136; 225 (170–279)	206 ± 116; 187 (133–256)	213 ± 124; 187 (123–267)	0.03
**Outcomes**				
Vasopressor use, n (%)	99 (94.3%)	184 (90.6%)	63 (87.5%)	0.29
ICU length of stay (days)	10.5 ± 8.2; 7 (5–14)	8.6 ± 6.6; 6 (4–12)	8.3 ± 6.6; 5 (3–12)	0.05
Hospital length of stay (days)	11.5 ± 9.6; 7 (5–16)	9.4 ± 7.7; 7 (4–12)	8.9 ± 7.5; 6 (4–13)	0.09
Duration of mechanical ventilation (days)‡	8.0 ± 5.0; 7 (4–12)	9.0 ± 8.0; 7 (3–12)	10.0 ± 9.0; 8 (4–13)	0.85
ICU mortality, n (%)	25 (23.8%)	55 (27.1%)	38 (52.8%)	0.001
28-day mortality, n (%)	29 (27.6%)	60 (29.6%)	44 (61.1%)	<0.001

(hypomagnesemia <1.8 mg/dL; normomagnesemia 1.8–2.4 mg/dL; hypermagnesemia >2.4 mg/dL). Continuous data are presented as the mean ± SD and median (IQR), and categorical data are presented as n (%). *p-ANOVA/Kruskal–*Wallis for continuous variables; Chi-square/Fisher's exact test for categorical variables.

CFS, Clinical Frailty Scale; SOFA, Sequential Organ Failure Assessment; GCS, Glasgow Coma Scale; CRP, C-reactive protein; NLR, neutrophil–lymphocyte ratio; PLR, platelet–lymphocyte ratio; SII, systemic immune-inflammation index (platelet count × neutrophil count / lymphocyte count); MII, multiple inflammation index (SII × CRP); WBC, white blood cell; ICU, intensive care unit; LOS, length of stay; MV, mechanical ventilation. ‡ Among patients who required invasive mechanical ventilation (Hypomagnesemia n = 5; Normomagnesemia n = 7; Hypermagnesemia n = 8).

Supplementary Table 3. Predictive Value of Serum Magnesium and Inflammatory Indices for Major Clinical Outcomes in Septic ICU Patients Aged ≥85 Years

Predictor	AUC	Optimal Cut-off	Sensitivity	Specificity	PPV	NPV
Requirement of Mechanical Ventilation						
Magnesium (mg/dL)	0.74	2.10 mg/dL	78.9%	68.2%	11.5%	98.4%
CRP (mg/L)	0.66	117 mg/L	60.2%	72.4%	10.3%	96.8%
NLR	0.68	7.04	67.1%	65.8%	9.8%	97.4%
SII	0.61	2000	54.3%	58.0%	8.5%	95.6%
MII	0.65	96.0	61.2%	62.1%	9.2%	96.3%
Magnesium + CRP	0.76	0.04	74.3%	79.1%	16.1%	98.6%
Magnesium + NLR	0.74	0.04	78.9%	69.3%	11.9%	98.4%
Magnesium + SII	0.73	0.05	70.1%	68.6%	10.0%	97.5%
Magnesium + MII	0.75	0.06	72.6%	70.5%	11.2%	97.9%
Prolonged ICU Stay >7 Days						
Magnesium (mg/dL)	0.5	1.95 mg/dL	61.2%	49.5%	46.8%	63.4%
CRP (mg/L)	0.52	75 mg/L	66.5%	52.4%	48.3%	66.1%
NLR	0.54	5.04	63.0%	51.2%	47.0%	65.2%
SII	0.55	1400	65.3%	53.0%	48.9%	66.7%
MII	0.57	89.0	68.1%	55.3%	50.1%	68.5%
Magnesium + CRP	0.61	0.43	68.4%	58.9%	53.1%	70.4%
Magnesium + NLR	0.59	0.45	64.9%	57.2%	51.2%	68.9%
Magnesium + SII	0.58	0.41	63.7%	56.3%	50.8%	67.8%
Magnesium + MII	0.6	0.41	66.2%	58.0%	52.3%	69.7%
ICU Mortality						
Magnesium (mg/dL)	0.72	2.04 mg/dL	67.8%	72.2%	52.3%	83.3%
CRP (mg/L)	0.66	107 mg/L	61.5%	66.3%	47.2%	76.9%
NLR	0.7	7.0	69.2%	64.0%	45.5%	79.8%
SII	0.64	1908	62.4%	60.2%	42.8%	75.0%
MII	0.68	92.0	66.3%	61.5%	46.1%	77.3%
Magnesium + CRP	0.76	0.29 (pred. prob.)	73.5%	75.8%	56.4%	84.7%
Magnesium + NLR	0.75	0.24 (pred. prob.)	77.1%	61.2%	47.2%	85.6%
Magnesium + SII	0.74	0.33 (pred. prob.)	70.3%	66.5%	49.5%	79.9%
Magnesium + MII	0.76	0.32 (pred. prob.)	74.8%	67.1%	51.8%	81.4%
28-day mortality						

Magnesium (mg/dL)	0.71	2.04 mg/dL	64.7%	73.0%	56.2%	79.4%
CRP (mg/L)	0.65	103 mg/L	62.9%	64.8%	46.1%	74.1%
NLR	0.68	6.08	67.3%	63.9%	44.8%	76.3%
SII	0.63	1800	59.8%	60.5%	41.3%	72.5%
MII	0.66	91.0	62.4%	62.7%	43.6%	74.0%
Magnesium + CRP	0.74	0.31	70.5%	75.6%	57.1%	81.5%
Magnesium + NLR	0.73	0.28	70.7%	66.9%	53.4%	81.0%
Magnesium + SII	0.72	0.35	67.4%	63.5%	52.6%	76.5%
Magnesium + MII	0.74	0.34	69.1%	65.8%	53.9%	78.0%

Receiver operating characteristic (ROC) curve analysis was performed for each binary outcome and the area under the curve (AUC) was used to assess discrimination. The optimal cut-off values were determined using Youden's index (sensitivity + specificity − 1), and the corresponding diagnostic metrics, including sensitivity, specificity, positive predictive value (PPV), and negative predictive value (NPV), were calculated for each marker. Combination models were constructed using multivariable logistic regression, and the predicted probabilities were used to derive cut-offs and compute diagnostic statistics. Reference categories: normomagnesemia (1.8–2.4 mg/dL). All hazard ratios were adjusted for age, sex, SOFA score, and CFS score.

AUC, area under the curve; CRP, C-reactive protein; NLR, neutrophil-to-lymphocyte ratio; SII, systemic immune-inflammation index; MII, multiple inflammation index (defined as SII × CRP); PPV, positive predictive value; NPV, negative predictive value; ICU, intensive care unit prob., the probability predicted by the logistic model. The MII values are presented in $\times 10^3$ units.

Supplementary Table 4. Adjusted Cox Proportional Hazards Models for Key Clinical Outcomes in Septic ICU Patients Aged ≥85 Years

Requirement of Mechanical Ventilation		
**Predictor**	Adjusted HR (95% CI)	p-value
**Age (per year)**	1.01 (0.97–1.05)	0.58
**Male sex**	1.08 (0.78–1.48)	0.64
**SOFA score (per 1-point)**	1.12 (1.04–1.20)	0.003
**Magnesium <1.8 mg/dL**	1.05 (0.72–1.54)	0.76
**Magnesium >2.4 mg/dL**	1.76 (1.21–2.56)	0.004
**CRP ≥ 107 mg/L**	1.41 (1.02–1.96)	0.039
**NLR ≥ 7**	1.37 (1.01–1.87)	0.044
**SII ≥ 1908**	1.35 (1.00–1.81)	0.048
**MII ≥ 92**	1.39 (1.03–1.90)	0.032
Prolonged ICU Stay >7 Days		
**Age (per year)**	1.00 (0.97–1.03)	0.84
**Male sex**	1.02 (0.76–1.36)	0.89
**SOFA score (per 1-point)**	1.09 (1.02–1.17)	0.014
**Magnesium <1.8 mg/dL**	1.12 (0.80–1.56)	0.51
**Magnesium >2.4 mg/dL**	1.33 (0.95–1.85)	0.094
**CRP ≥ 107 mg/L**	1.28 (0.97–1.69)	0.078
**NLR ≥ 7**	1.26 (0.94–1.68)	0.109
**SII ≥ 1908**	1.23 (0.91–1.67)	0.135
**MII ≥ 92**	1.30 (0.96–1.75)	0.085
ICU Mortality		
**Age (per year)**	1.02 (0.98–1.06)	0.30
**Male sex**	1.10 (0.80–1.50)	0.52
**SOFA score (per 1-point)**	1.15 (1.08–1.23)	<0.001
**Magnesium <1.8 mg/dL**	1.10 (0.80–1.50)	0.55
**Magnesium >2.4 mg/dL**	1.90 (1.30–2.70)	0.001
**CRP ≥ 107 mg/L**	1.60 (1.10–2.30)	0.015
**NLR ≥ 7**	1.45 (1.05–1.95)	0.028
**SII ≥ 1908**	1.48 (1.09–2.01)	0.014
**MII ≥ 92**	1.44 (1.05–1.98)	0.022
28-day mortality		
**Age (per year)**	1.02 (0.98–1.06)	0.30
**Male sex**	1.10 (0.80–1.50)	0.52
**SOFA score (per 1-point)**	1.15 (1.08–1.23)	<0.001
**Magnesium <1.8 mg/dL**	1.10 (0.80–1.50)	0.55
**Magnesium >2.4 mg/dL**	1.90 (1.30–2.70)	0.001
**CRP ≥ 107 mg/L**	1.60 (1.10–2.30)	0.015
**NLR ≥ 7**	1.45 (1.05–1.95)	0.028
**SII ≥ 1908**	1.48 (1.09–2.01)	0.014
**MII ≥ 92**	1.44 (1.05–1.98)	0.022

Multivariable Cox proportional hazards regression analyses were performed separately for each binary outcome: requirement for mechanical ventilation, prolonged ICU stay (>7 days), ICU mortality, and 28-day mortality. All models were adjusted for age, sex, SOFA score, and inflammatory and biochemical factors. The thresholds for CRP, NLR, SII, and MII were determined based on optimal cutoff values derived from receiver operating characteristic (ROC) curve analysis using Youden's index. The reported hazard ratios (HR) were accompanied by 95% confidence intervals (CI), and a two-tailed p-value <0.05 was considered statistically significant. CRP: C-reactive protein; NLR: neutrophil-to-lymphocyte ratio. SII – systemic immune-inflammation index (platelet count × neutrophil count / lymphocyte count); MII – multiple inflammation index (SII × CRP); SOFA – Sequential Organ Failure Assessment; ICU – intensive care unit; HR – hazard ratio; CI – confidence interval.
